# Novel gain-of-function mutation of *TRPV4* associated with accelerated chondrogenic differentiation of dental pulp stem cells derived from a patient with metatropic dysplasia

**DOI:** 10.1016/j.bbrep.2019.100648

**Published:** 2019-05-17

**Authors:** Kentaro Nonaka, Xu Han, Hiroki Kato, Hiroshi Sato, Haruyoshi Yamaza, Yuta Hirofuji, Keiji Masuda

**Affiliations:** a Department of Surgery and Science, Graduate School of Medical Sciences, Kyushu University, Maidashi 3-1-1, Higashi-Ku, Fukuoka, 812-8582, Japan; b Section of Oral Medicine for Children, Division of Oral Health, Growth and Development, Faculty of Dental Science, Kyushu University, Maidashi 3-1-1, Higashi-Ku, Fukuoka, 812-8582, Japan

**Keywords:** Chondrocyte differentiation, Dental pulp stem cell, Metatropic dysplasia, Transient receptor potential vanilloid 4, 4αPDD, 4α-phorbol 12,13-didecanoate, CC, chondrocyte, DPSC, dental pulp stem cell, MD, metatropic dysplasia, SOX9, SRY-box 9, TRPV4, transient receptor potential vanilloid 4

## Abstract

Metatropic dysplasia is a congenital skeletal dysplasia characterized by severe platyspondyly, dumbbell-like deformity of long tubular bones, and progressive kyphoscoliosis with growth. It is caused by mutations in the gene *TRPV4*, encoding the transient receptor potential vanilloid 4, which acts as a calcium channel. Many heterozygous single base mutations of this gene have been associated with the disorder, showing autosomal dominant inheritance. Although abnormal endochondral ossification has been observed by histological examination of bone in a patient with lethal metatropic dysplasia, the etiology of the disorder remains largely unresolved. As dental pulp stem cells (DPSCs) are mesenchymal stem cells that differentiate into bone lineage cells, DPSCs derived from patients with congenital skeletal dysplasia might be useful as a disease-specific cellular model for etiological investigation. The purpose of this study was to clarify the pathological association between *TRPV4* mutation and chondrocyte differentiation by analyzing DPSCs from a patient with non-lethal metatropic dysplasia. We identified a novel heterozygous single base mutation, c.1855C>T in *TRPV4*. This was predicted to be a missense mutation, p.L619F, in putative transmembrane segment 5. The mutation was repaired by CRISPR/Cas9 system to obtain isogenic control DPSCs for further analysis. The expression of stem cell markers and fibroblast-like morphology were comparable between patient-derived mutant and control DPSCs, although expression of TRPV4 was lower in mutant DPSCs than control DPSCs. Despite the lower TRPV4 expression in mutant DPSCs, the intracellular Ca^2+^ level was comparable at the basal level between mutant and control DPSCs, while its level was markedly higher following stimulation with 4α-phorbol 12,13-didecanoate (4αPDD), a specific agonist for TRPV4, in mutant DPSCs than in control DPSCs. In the presence of 4αPDD, we observed accelerated early chondrocyte differentiation and upregulated mRNA expression of SRY-box 9 (*SOX9*) in mutant DPSCs. Our findings suggested that the novel missense mutation c.1855C>T of *TRPV4* was a gain-of-function mutation leading to enhanced intracellular Ca^2+^ level, which was associated with accelerated chondrocyte differentiation and *SOX9* upregulation. Our results also suggest that patient-derived DPSCs can be a useful disease-specific cellular model for elucidating the pathological mechanism of metatropic dysplasia.

## Highlights

• Dental pulp stem cells from a patient with metatropic dysplasia were analyzed.
• A novel heterozygous single base mutation, c.1855C > T, was identified in *TRPV4.*
• The mutation was repaired by gene editing to obtain isogenic control cells.
• The intracellular Ca^2+^ level was enhanced in mutant cells by 4αPDD.
• Chondrocyte differentiation was enhanced in mutant cells by 4αPDD.

## 1 Introduction

Metatropic dysplasia (MD) is one of several skeletal dysplasias caused by heterozygous mutations in *TRPV4*, a gene encoding the transient receptor potential vanilloid 4 [1,2]. Various degrees of severity have been reported for non-lethal MD, and the disorder can be lethal [[3], [4], [5]]. In the non-lethal type, the limbs are shorter than the trunk at birth, and progressive kyphoscoliosis worsens this condition during growth [1]. Histological examination and the core radiographic findings including severe platyspondyly and dumbbell-shaped deformity of tubular bones suggest dysregulated endochondral ossification associated with dysfunction of TRPV4 [4].

Functional TRPV4 is activated by various noxious stimuli and can form homo- or hetero-tetramers on the cell membrane [6,7]. The ectopic expression of exclusively mutant TRPV4 in cell lines results in enhanced Ca^2+^ influx [3,4]. However, considering that heterozygous mutations of *TRPV4* cause MD, functional TRPV4 may be formed at various stoichiometric ratios of mutant and wild-type TRPV4 monomers in patients [2]. In addition, TRPV4-deficient mice have not shown spinal or tubular bone abnormalities [8,9]. The mechanism of pathogenic dysfunction of TRPV4 channels in MD therefore remains unclear.

Cellular models derived from patients may be useful tools to analyze the genotype–phenotype correlations of MD, but only a few studies have been reported [10,11]. Human dental pulp stem cells (DPSCs) are mesenchymal stem cells that have been shown to differentiate into bone lineage cells [12,13]. DPSCs derived from patients with congenital skeletal dysplasia are, therefore, likely to be a useful disease-specific cellular model for investigating the underlying pathology. The purpose of this study was to elucidate the association between *TRPV4* mutation and chondrogenic differentiation in MD through genetic and cell biological analyses of DPSCs derived from a patient with non-lethal MD. Our results suggested that a novel gain-of-function mutation in *TRPV4* was associated with a markedly increased intracellular Ca^2+^, SRY-box 9 (*SOX9*) upregulation and accelerated chondrocyte differentiation in response to 4α-phorbol 12,13-didecanoate (4αPDD).

## 2 Materials and methods

### 2.1 Isolation and culture of DPSCs

Experiments using human samples were reviewed and approved by the Kyushu University Institutional Review Board for Human Genome/Gene Research (permission number: 678–00), and were conducted in accordance with the Declaration of Helsinki. Dental pulp was isolated from a 14-year-old patient with MD. Written informed consent was obtained from the patient's guardians. The dental pulp was treated with 3 mg/mL collagenase I (Worthington, NJ, USA) and 4 mg/mL dispase II (Wako, Osaka, Japan) in phosphate-buffered saline (PBS) containing 2 mM CaCl_2_ for 60 min at 37 °C. Next, the undigested tissues and cell aggregates were removed by filtration through a 70-μm cell strainer (Corning, NY, USA). Single-cell suspensions were then seeded in a 6-well plate (Corning) and cultured in medium consisting of Alpha Modification of Eagle's Medium (αMEM; Sigma-Aldrich, MO, USA) containing 15% fetal bovine serum (Sigma-Aldrich), 100 μM l-ascorbic acid 2-phosphate (Wako), 2 mM l-glutamine (Life Technologies, NY, USA), 250 μg/mL fungizone (Life Technologies), 100 U/mL penicillin, and 100 μg/mL streptomycin (Life Technologies), at 37 °C, in an atmosphere containing 5% CO_2_. Cells passaged seven times or less were used in all experiments.

### 2.2 DNA sequence and mutation analysis

Total RNA was extracted from cells using the RNeasy Mini Kit (Qiagen, Hilden, Germany). For cDNA reverse transcription, 5 μg total RNA was reverse transcribed using SuperScript III First-Strand Synthesis System (Life Technologies) and primed with oligo (dT) according to the manufacturer's instructions. We amplified *TRPV4* cDNA by PCR using PrimeStar HS DNA polymerase (Takara, Shiga, Japan) from this first-strand cDNA. The primer sequences were as follows: forward primer 5′-GCTGAGCAGTGCAGACGGGCCT-3′; reverse primer 5′-CATTTATTGAGCACCGGCAAATC-3′. The amplified PCR product was sequenced by Sanger method, and sequences were aligned to an Ensembl *TRPV4* reference sequence (ENST00000261740.6; Chromosome 12q24.11). Heterozygous changes were compared with those in the NCBI SNP database.

### 2.3 Flowcytometric analysis

Cells were cultured in a 10-cm culture dish (Corning) at 70% confluency, and treated with accutase (Nacalai Tesque, Kyoto, Japan) to detach them from the culture dish. The cells were then fixed with 2% paraformaldehyde in PBS for 15 min, and blocked with 1% BSA (Wako) in PBS for 20 min. Subsequently, the cells were incubated (1 × 10^6^ cells per 100 μL) with PE-labeled anti-human CD90 (#BL328110, BioLegend, CA, USA), anti-human CD73 (#BL344004, BioLegend), anti-human CD34 (#BL343506, BioLegend), and mouse IgG1 kappa isotype control antibodies (#BL400114, BioLegend) for 1 h at room temperature in dark. Populations of 10,000 cells were analyzed using FACSVerse (BD Bioscience, CA, USA) and FACSuite software (BD Bioscience).

### 2.4 Western blot analysis

We lysed the cells with sample buffer (62.5 mM Tris-HCl buffer [pH 6.8] containing 2% sodium dodecyl sulfate [SDS], 5% β-mercaptoethanol, and 10% glycerol). The cell lysates were incubated at 95 °C and analyzed by SDS-polyacrylamide gel electrophoresis. Subsequently, immunoblotting was performed using *anti*-TRPV4 (#ACC-034, Alomone labs, Jerusalem, Israel) and α-tubulin (#sc-32293, Santa Cruz Biotechnology, CA, USA) antibodies. The membranes were washed and incubated with HRP-conjugated secondary antibody (for TRPV4: #sc-2004 or for α-tubulin: sc-2005, Santa Cruz Biotechnology) for 1 h at room temperature and visualized with ECL prime (GE Healthcare, Buckinghamshire, UK). The signals were detected and quantified using LAS-1000 pro (Fuji Film, Tokyo, Japan) with Image Gauge software (Fuji Film). The expression of TRPV4 was normalized to α-tubulin protein levels.

### 2.5 Immunocytochemistry

Cells cultured on coverslips were fixed with methanol for 10 min at −20 °C, blocked with 2% BSA (Wako) in PBS for 20 min, and then incubated with *anti*-TRPV4 antibody (#ACC-034, Alomone labs). After 90 min, they were incubated with Alexa Fluor 568-conjugated secondary antibody (#A11012, Life Technologies) in dark for 60 min. Following antibody staining, the nuclei were stained with 1 μg/mL 4′,6-diamidino-2-phenylindole dihydrochloride (DAPI; Dojindo, Kumamoto, Japan), and mounted using ProLong Diamond (Life Technologies). Fluorescent images were captured using a Nikon C2 confocal microscope (Nikon, Tokyo, Japan). The images were analyzed using NIS-Elements software version 4.00.06 (Nikon).

### 2.6 Chondrogenic differentiation

Chondrogenic differentiation was performed using micro-mass culture system as previously described with slight modifications [14]. The cells were detached from their dish using Trypsin/EDTA (Nacalai Tesque) and resuspended in chondrogenic differentiation medium consisting of culture medium with 10 ng/mL transforming growth factor beta-3 (TGFβ3; PeproTech, NJ, USA), 100 nM dexamethasone (Sigma-Aldrich), 1% insulin-transferrin-selenium mixture (Life Technologies) and 1 μM 4α-phorbol 12,13-didecanoate (4αPDD; Wako) at a concentration of 2 × 10^6^ cells/mL. Four spots of 10 μL micro-mass were placed in a well of 12-well tissue culture plates. After 3 h, 1 mL of chondrogenic differentiation medium was added gently. The cells were then cultured for seven days at 37 °C in an atmosphere containing 5% CO_2_. The chondrogenic differentiation medium was changed every three days.

### 2.7 Alcian blue staining

The cells were fixed with 2% acetic acid in ethanol for 15 min at room temperature and then rehydrated by soaking in 95% and 70% ethanol for 10 min. They were then stained with Alcian blue stain solution (Nacalai Tesque) for 16 h at 4 °C. To quantify Alcian blue staining, Alcian blue was extracted from the cells by incubation with 6 M guanidine hydrochloride for 16 h at room temperature, and absorbance was measured at 595 nm using an Infinite 200 PRO plate reader (Tecan, Männedorf, Switzerland).

### 2.8 RNA extraction and quantitative real-time polymerase chain reaction (RT-qPCR)

Total RNA was extracted from the cells using an RNAeasy Mini Kit (Qiagen). First-strand cDNA was synthesized using the ReverTra Ace qPCR RT Master Mix with gDNA Remover (Toyobo, Osaka, Japan). The sequences of primers used were as follows: *SOX9*: 5′- GGAAGTCGGTGAAGAACGGG-3′ (forward), 5′- CTCGCTTCAGGTCAGCCTTG-3′ (reverse); *RPL13A*: 5′- GCTGTGAAGGCATCAACATTT-3 (forward), 5′- CATCCGCTTTTTCTTGTCGTA-3′ (reverse). Real-time quantitative PCR was performed using the GoTaq qPCR Master Mix (Promega, WI, USA) and analyzed with the StepOnePlus Real-Time PCR System (Life Technologies). The threshold cycle (Ct) value of *RPL13A* (housekeeping gene) was subtracted from the Ct value of *SOX9* (ΔCt). The relative expression of *SOX9* is shown as fold changes determined using the 2^−ΔΔCt^ method.

### 2.9 Gene editing

Repair of the single nucleotide substitution c.1855C>T in *TRPV4* was performed using CRISPR/Cas9 and single-stranded oligodeoxynucleotide (ssODN) repair template. Guide RNA (sgRNA) specifically targeting the mutation site of *TRPV4* mutation allele was designed using CRISPRdirect software (http://crispr.dbcls.jp/). A site not included in the normal genomic sequence was picked. The target sequence was as follows: 5′-TGAAGAGCAAGTAGACGAAC -3′. The sgRNA was generated using the Guide-it sgRNA In Vitro Transcription Kit (Takara) according to the manufacturer's instructions. To generate a ribonucleoprotein (RNP) complex, 9.9 μg of recombinant Streptococcus pyogenes Cas9 protein (SpCas9, Takara) and 2 μg of sgRNA were mixed and incubated at 37 °C for 5 min. A 150-nt ssODN was manufactured by Eurofins Genomics (Tokyo, Japan). The sequence of the ssODN sequence was as follows: 5′-ATGTGTCTCCTCTTTGCCTCCATAATCCCGCTGGGGTCTTTAGATTCTCTTCAAGGACCTTTTCCGATTCCTGCTCGTCTACTTGCTCTTCATGATCGGCTACGCTTCAGGTGAGCTCTGGGTGCTCAGGTGGTCCTGGCAGGGGTGGTG-3′.

The cells were detached from their dish using trypsin/EDTA and suspended in Opti-MEM (Life Technologies). We then electroporated 1 × 10^6^ cells with the RNP complex and ssODN (150 pmols) using a super electroporator NEPA21 TypeII (Nepa Gene, Chiba, Japan) and EC-002S NEPA electroporation cuvettes (2 mm gap; Nepa Gene). For poring pulse, voltage, length, interval, number, decay rate, and polarity were set as 150 V, 5 ms, 50 ms, 2 times, 10%, and ‘+’, respectively. For transfer pulse, voltage, length, interval, number, decay rate, and polarity were set as 20 V, 50 ms, 50 ms, 5 times, 40%, and ‘+/−’, respectively. After culture for three days on a 10-cm dish, the cells were plated at limiting dilution in 96-well plates, and cell clones were obtained. These clones were passaged on 24-well plates. Genomic DNA was then isolated from some cells, and the remaining cells were preserved. To select the clones with repaired *TRPV4*, negative screening was carried out using PCR that specifically amplifies a mutant DNA fragment from genomic template DNA and sequencing as described above (section 2.2). The primer sequences were as follows: forward primer 5′-CAAGGACCTTTTCCGATTCCAGT-3′; reverse primer 5′- GGCAAGGATGGAAACGTACTTAGTC-3′.

### 2.10 Intracellular calcium measurement

To measure intracellular calcium, the cells were cultured in 96-well plates and stained with 10 μM Fura-2 AM (Dojindo), 0.05% (w/v) Pluronic F-127 (Sigma-Aldrich), and 500 μM probenecid (Sigma-Aldrich) in Hanks' Balanced Salt Solution (HBSS) for 45 min at 37 °C. The fluorescence signals were measured by excitation at 340 and 380 nm and emission at 510 nm using an EnSight plate reader (PerkinElmer, MA, USA) at 37 °C. For stimulation of TRPV4, 1 μM 4αPDD (Wako) was added.

### 2.11 Statistical analyses

Statistical analyses were performed by Student's *t*-test using JMP software version 12 (SAS Institute, NC, USA). Results with P values < 0.05 were considered significant.

## 3 Results

### 3.1 Clinical and radiographic features and TRPV4 mutation in the patient

**Fig. 1:**
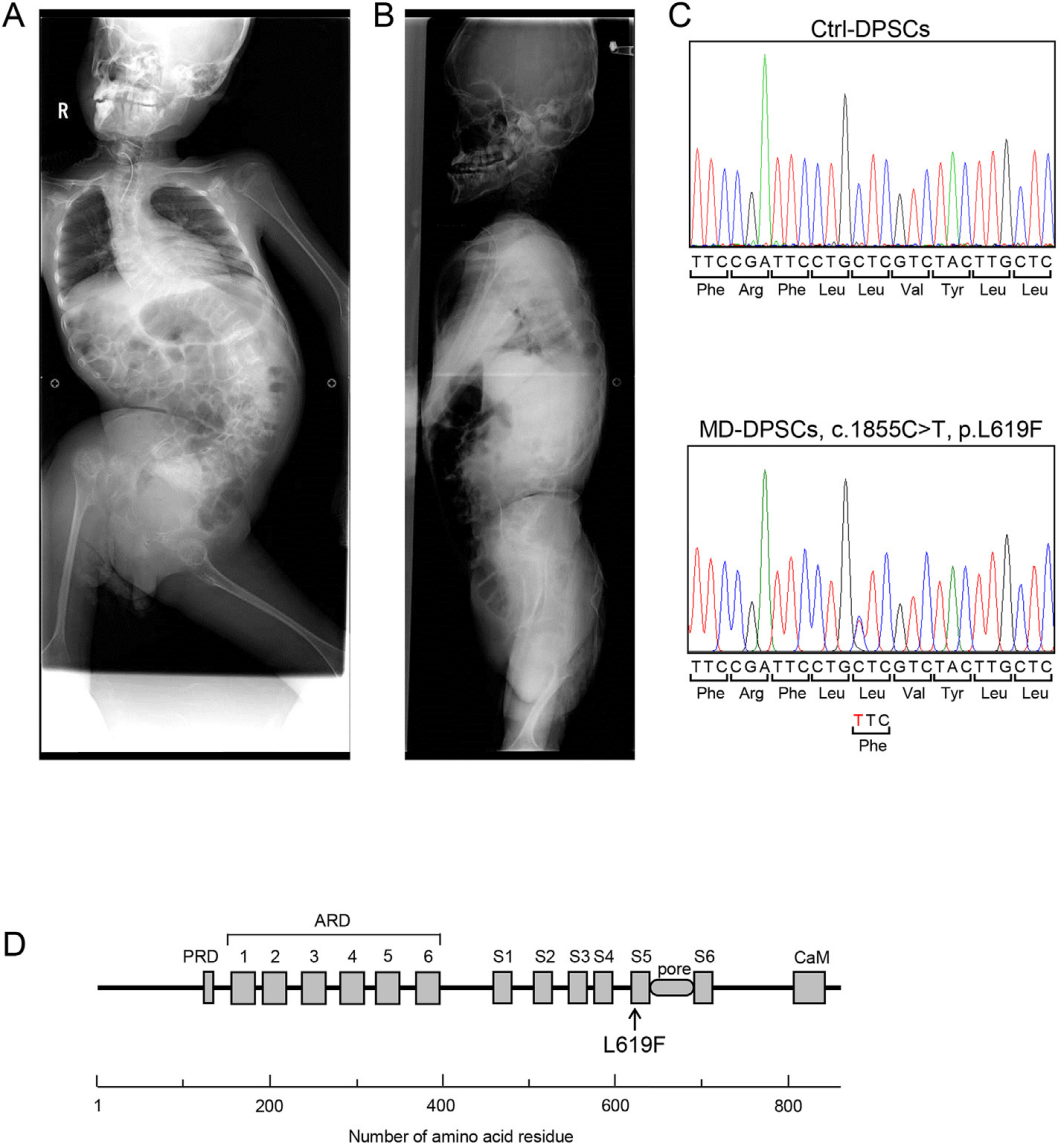
Novel mutation of the *TRPV4* gene in a patient with MD. (A, B) Anterior (A) and lateral (B) views of radiographs of the patient with MD studied here. (C) DNA sequence of *TRPV4* from Ctrl- and MD-DPSCs. (D) Schematic diagram of TRPV4's predicted structure and the position of the mutation identified in this study. PRD, proline-rich domain; ARD, ankyrin repeat domain; S1–S6, transmembrane segments; pore, Ca^2+^ pore domain; CaM, calmodulin binding motif.

The patient was a 14-year-old boy, who was referred to our department with toothache. He was diagnosed with MD in view of skeletal manifestations after birth. There was no family history of skeletal dysplasia. He had difficulty walking. At 11 years of age, surgical treatment was performed for atlantoaxial subluxation with odontoid hypoplasia. The radiographic findings at 14 years of age included severe kyphoscoliosis, platyspondyly, and knobby joints (Fig. 1A and B).

Mutation analysis against Ensembl *TRPV4* reference sequence ENST00000261740.6 identified a heterozygous single base mutation, c.1855C>T, which was predicted to be p.L619F in the putative transmembrane segment 5 (S5) of TRPV4 (Fig. 1C and D). This mutation was not found in the NCBI dbSNP, Ensembl SNP, and EVS (Exome Variant Server) databases, suggesting a novel dominant missense mutation of *TRPV4* associated with non-lethal MD.

### 3.2 Surface phenotypes and TRPV4 expression of patient-derived DPSCs

**Fig. 2:**
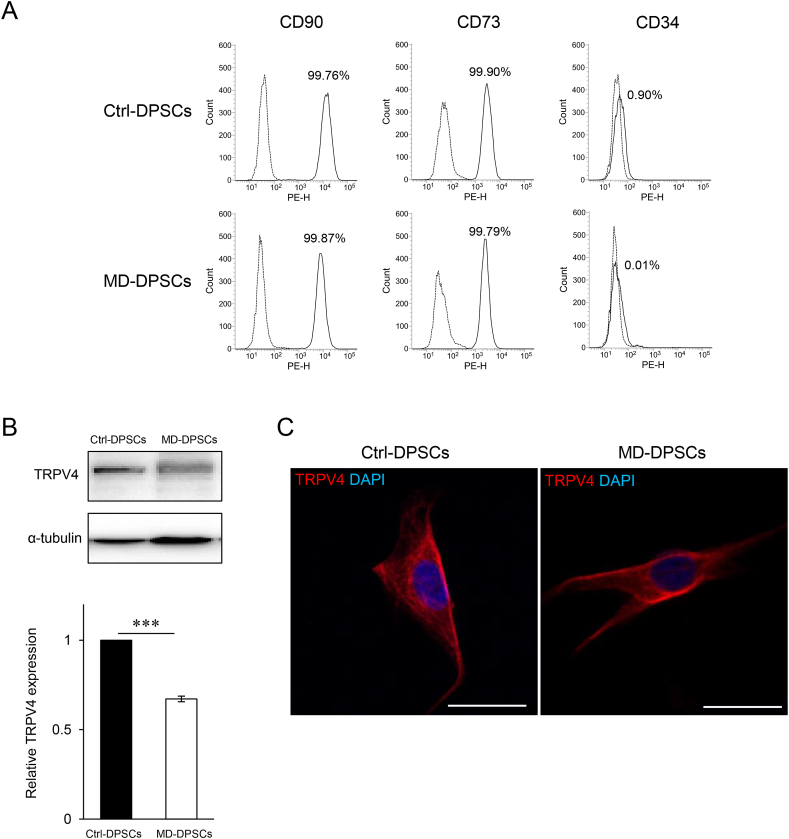
Characteristics of MD-DPSCs. (A) Flow cytometry analysis of stem cell markers in DPSCs. The solid line histogram plots CD90, CD73 (mesenchymal stem cell marker), and CD34 (hematopoietic stem cell marker). The dot-line histogram shows a plot of isotype control. Each number (%) indicates the proportion of positive cells. (B, C) The expression of TRPV4 analyzed with western blotting (B) and immunocytostaining (C). Scale bar = 25 μm. The graph in (B) represents the mean ± standard deviation (SD) from two independent experiments, one of which included two technical replicates, and the other was performed only once. ***P < 0.001.

The expression profiles of stem cell markers CD90, CD73, and CD34 in DPSCs with mutant TRPV4 derived from this patient (MD-DPSCs) were comparable to those in isogenic control DPSCs in which c.1855C>T in MD-DPSCs was corrected by CRISPR/Cas9 (Ctrl-DPSCs) (Fig. 1, Fig. 2A). Western blot analysis showed reduced expression of TRPV4 in MD-DPSCs compared with that in Ctrl-DPSCs (Fig. 2B). Immunofluorescent staining of TRPV4 showed a similar pattern between MD-DPSCs and Ctrl-DPSCs, although the cell surface and subcellular localization of TRPV4 were not clearly determined (Fig. 2C and Supplemental Fig. A). Fibroblast-like morphology was comparable to that of Ctrl-DPSCs (Supplemental Fig. B). These results suggested that mutant TRPV4 did not alter the characteristics of DPSCs, although it might affect protein expression levels.

### 3.3 Increased intracellular Ca^2+^ level in MD-DPSCs

**Fig. 3:**
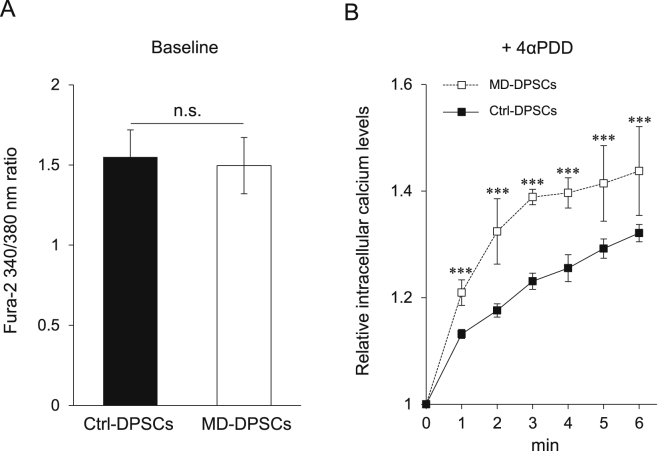
Altered calcium homeostasis in MD-DPSCs. (A) Basal intracellular calcium levels in DPSCs. The graph represents the mean ± standard deviation (SD) of 340/380 nm fura-2 fluorescence ratio from three independent experiments, each consisting of four technical replicates. n.s., not significant. (B) Relative intracellular calcium levels after activation of TRPV4 by 1 μM 4αPDD in DPSCs. The intracellular calcium levels were measured every minute for 6 min after addition of 4αPDD. The 340/380 nm fura-2 fluorescence ratio at time 0 in each DPSC sample was set as 1. The graph represents mean ± SD from four technical replicates. ***P < 0.001.

Various mutations of *TRPV4* associated with skeletal dysplasia have been reported to alter the Ca^2+^ channel function of TRPV4. In the present study, the baseline intracellular Ca^2+^ level was similar between MD-DPSCs and Ctrl-DPSCs (Fig. 3A). In contrast, when stimulated with 4αPDD, the intracellular Ca^2+^ level in MD-DPSCs was significantly and persistently elevated in comparison to that in Ctrl-DPSCs (Fig. 3B). These results suggested that the heterozygous mutation c.1855C>T (p.L619F) was associated with enhanced TRPV4 channel function, resulting in dysregulation of intracellular Ca^2+^ levels in response to 4αPDD.

### 3.4 Accelerated chondrogenic differentiation in MD-DPSCs

**Fig. 4:**
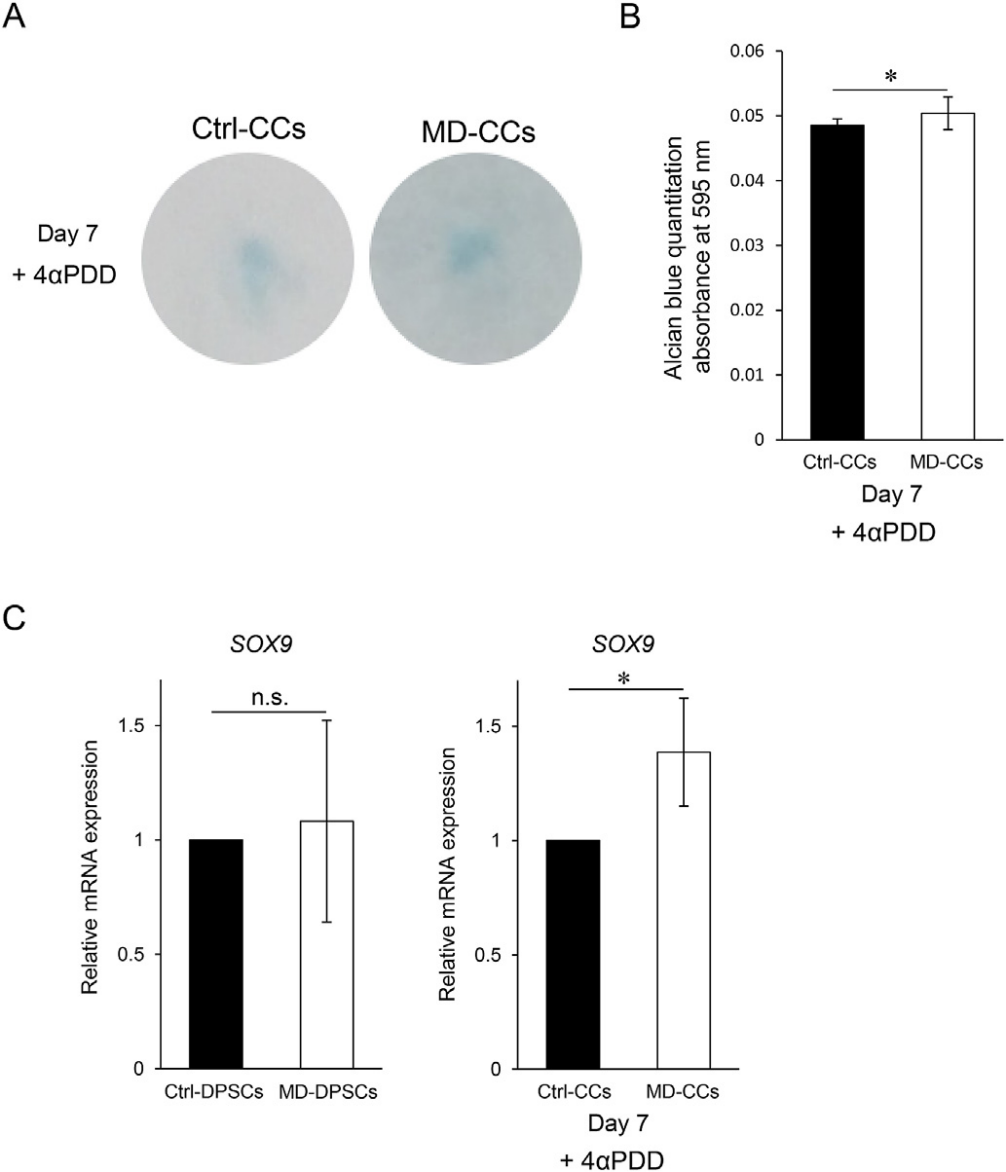
Accelerated chondrogenic differentiation of MD-DPSCs. (A) Alcian blue staining of CCs differentiated for seven days. (B) Alcian blue was extracted and measured by absorbance at 595 nm. The graph represents the mean ± SD from four technical replicates. *P < 0.05. (C) The relative mRNA expression of SOX9 in DPSCs (left panel) and CCs (right panel). The expression in isogenic control cells was set as one. Graphs show the mean ± SD from three technical replicates. n.s., not significant. *P < 0.05. (For interpretation of the references to colour in this figure legend, the reader is referred to the Web version of this article.)

The skeletal phenotype of MD is mainly characterized by abnormal endochondral ossification. We examined whether the enhanced TRPV4 channel function observed in MD-DPSCs affected chondrocyte differentiation *in vitro*. After differentiation into chondrocytes (CCs) in the presence of 4αPDD, the staining intensity of Alcian blue on day 7 was significantly higher in MD-CCs than in Ctrl-CCs (Fig. 4A and B). Consistent with this result, mRNA expression level of *SOX9* was significantly higher in MD-CCs than in Ctrl-CCs, although it was comparable between MD-DPSCs and Ctrl-DPSCs (Fig. 4C). These results suggested that the mutant TRPV4 might accelerate early chondrogenic differentiation and upregulate *SOX9*.

## 4 Discussion

In this study, we analyzed DPSCs obtained from a patient with non-lethal MD as a disease-specific cellular model. The patient carried a novel heterozygous single base mutation in *TRPV4*, c.1855C>T, which leads to a missense mutation, p.L619F. MD-DPSCs with this mutation were compared with isogenic Ctrl-DPSCs in which c.1855C>T was repaired by the CRISPR/Cas9 system. MD-DPSCs showed enhanced intracellular Ca^2+^ levels, accelerated chondrocyte differentiation and *SOX9* upregulation in response to 4αPDD, suggesting that p.L619F is a gain-of-function mutation.

Although patient-derived cells are useful models for elucidating the etiology of TRPV4-associated skeletal dysplasia, only a few studies in patient-derived cells have been reported, possibly because of the wide variety of genotypes and phenotypes [[1], [2], [3], [4], [5]]. Induced pluripotent stem cells derived from patients with lethal MD caused by mutation p.I604 M of TRPV4 showed abnormal chondrogenic marker expression [10]. Primary chondrocytes derived from patients with non-lethal MD caused by mutations p.G800D and p.P799L showed enhanced intracellular Ca^2+^ levels under various conditions [11]. In this study, we analyzed patient-derived MD-DPSCs carrying mutant TRPV4 with p.L619F, compared to isogenic control DPSCs with wild type TRPV4 repaired by the CRISPR/Cas9 system. The expression of stem cell markers was comparable between MD-DPSCs and Ctrl-DPSCs, while the expression of TRPV4 was lower in MD-DPSCs than in Ctrl-DPSCs. The p.L619F is close to the C-terminal rather than the N-terminal regulating TRPV4 distribution and localization in cells [15,16]. Our results suggest that p.L619F might have no significant effect on characteristics of DPSCs and a negative effect on the production of TRPV4 protein by unelucidated mechanism.

We found that intracellular Ca^2+^ levels in MD-DPSCs were clearly higher following 4αPDD treatment than that in Ctrl-DPSCs, suggesting enhanced TRPV4 channel function. Considering that the channel function is related to its expression level, similar levels of intracellular Ca^2+^ at baseline between MD-DPSCs and Ctrl-DPSCs may be associated with decreased expression of mutant TRPV4 in MD-DPSCs. Increased intracellular Ca^2+^ levels in response to 4αPDD of MD-DPSCs, despite the decreased TRPV4 expression, further support the hypothesis that p.L619F is gain-of-function mutation. We also found accelerated early chondrogenic differentiation and upregulated *SOX9* in MD-DPSCs. SOX9 is an essential factor regulating the expression of genes necessary for early-stage endochondral ossification [17,18]. It has been shown that TRPV4 upregulates SOX9 expression and chondrocyte differentiation through the Ca^2+^/calmodulin signaling pathway, suggesting that TRPV4 has an important role during endochondral ossification [19]. Our results suggest that p.L619F might enhance the channel function of TRPV4, resulting in dysregulation of intracellular Ca^2+^ levels and upregulation of SOX9 downstream of Ca^2+^/calmodulin signals, leading to abnormal initiation of endochondral ossification.

This study has notable limitations in relation to genotype-phenotype correlation in MD. First, the accelerated chondrogenic differentiation shown here could not fully explain the *in vivo* mechanism of skeletal dysplasia in this patient. Mutant TRPV4 may also have affected the differentiation and function of osteoclasts or osteoblasts during chondrogenesis [9,20,21]. Second, the molecular mechanism of enhanced TRPV4 channel function by p.L619F mutation remains unclear. To elucidate this, it is necessary to further investigate the response to physiological stimulation necessary for chondrogenesis, such as cell swelling, calcium oscillation, and ion currents other than those of Ca^2+^. Third, the pathological relevance between the p.L619F mutation and the decreased expression of mutant TRPV4 in MD-DPSCs remains unresolved. While Ctrl-DPSCs were derived from single clone during mutation repair, MD-DPSCs were heterogeneous, suggesting the possible effect of clonal variation on the phenotypes. Further analysis including clonal variation and cloned MD-DPSCs will be required to resolve this issue.

In conclusion, we identified a novel dominant mutation of TRPV4, c.1855C>T, using DPSCs derived from a patient with non-lethal MD as a disease-specific cellular model. Our findings suggest that this mutation may cause enhanced TRPV4 channel function as well as accelerated chondrocyte differentiation. In TRPV4-associated skeletal dysplasia, a wide variation in *TRPV4* mutations and symptoms have been reported, but there have been only a few patient-derived disease models for etiological investigation. Patient-derived DPSCs will be useful for further elucidating the pathogenesis of TRPV4-associated skeletal dysplasia.

## Conflicts of interest

The authors have no conflicts of interest to declare.

## Funding

This work was supported by the Takeda Science Foundation, Japan and the Kaibara Morikazu Medical Science Promotion Foundation, Japan.
