# Supplementary material for: Novel gain-of-function mutation of TRPV4 associated with accelerated chondrogenic differentiation of dental pulp stem cells derived from a patient with metatropic dysplasia
Source: Biochem Biophys Rep. 2019 May 17;19:100648. doi: 10.1016/j.bbrep.2019.100648 (PMC6709385; doi:10.1016/j.bbrep.2019.100648)
Supplement: Multimedia component 1 [file mmc1.pdf]

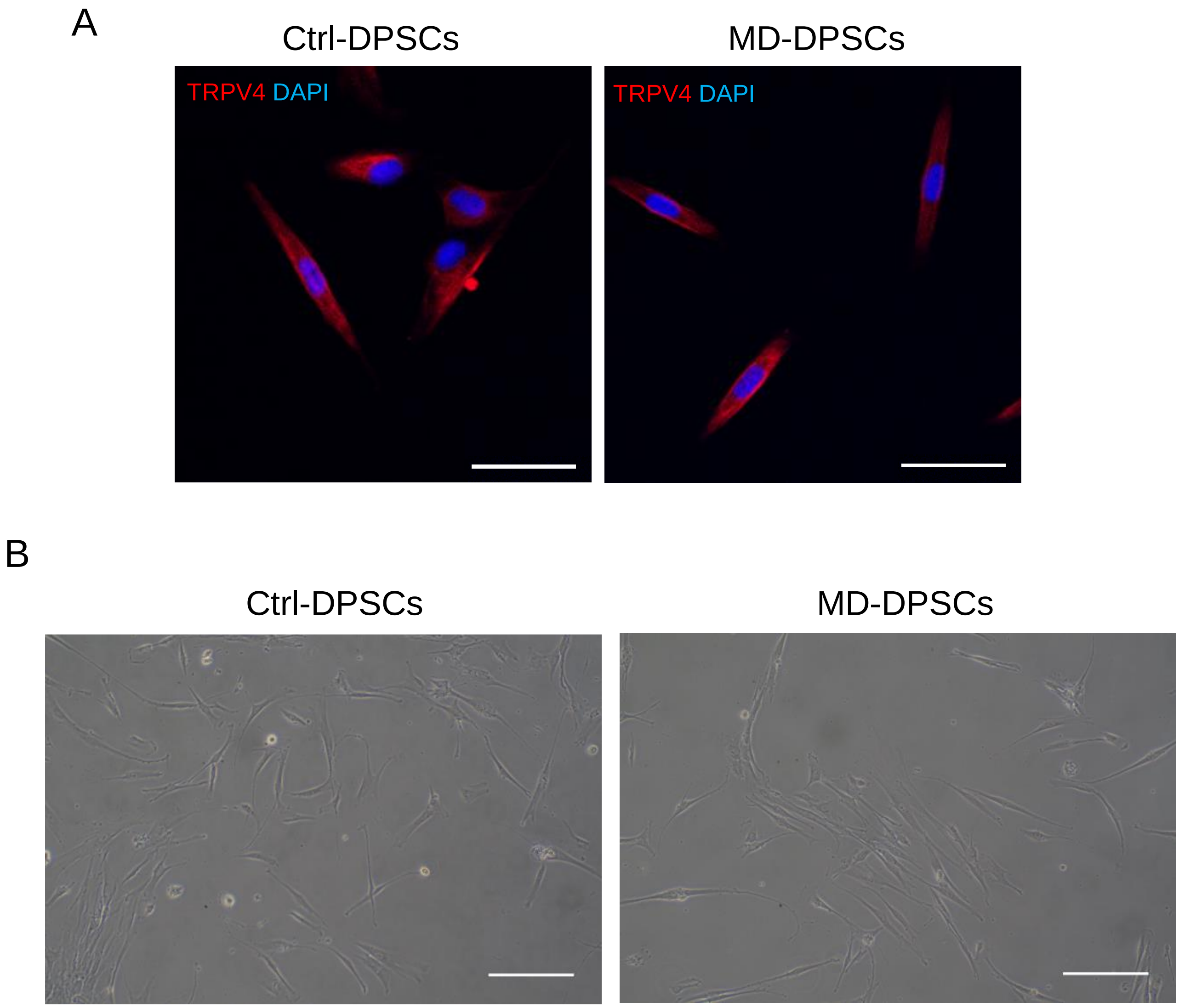

Supplemental figure. TRPV4 expression and morphology of DPSCs. (A) The expression of TRPV4 in DPSCs analyzed with immunocytostaining. Lower magnification of Fig.2C. Scale bar = 50 μm. (B) Morphology of DPSCs was observed using a phase-contrast microscope. Scale bar = 200 μm.

Supplemental Figure
